# Novel Mutations of *COL4A5* Identified in Chinese Families with X-Linked Alport Syndrome and Literature Review

**DOI:** 10.1155/2021/6664973

**Published:** 2021-03-02

**Authors:** Wen-yu Gong, Fan-na Liu, Liang-hong Yin, Jun Zhang

**Affiliations:** ^1^Division of Nephrology, Department of Medicine, The First Affiliated Hospital, Jinan University, Guangzhou, Guangdong 510630, China; ^2^Division of Nephrology, Department of Medicine, Third Affiliated Hospital of Sun Yat-Sen University, Guangzhou, Guangdong 510630, China

## Abstract

Alport syndrome (AS) is an inherited kidney disease caused by defects in type IV collagen, which is characterized by hematuria, progressive nephritis or end-stage renal disease (ESRD), hearing loss, and occasionally ocular lesions. Approximately 80% of AS cases are caused by X-linked mutations in the *COL4A5* gene. This study explored novel deletion and missense mutations in *COL4A5* responsible for renal disorder in two Han Chinese families. In pedigree 1, the five male patients all had ESRD at a young age, while the affected female members only presented with microscopic hematuria. Whole exome sequencing and Sanger sequencing identified a novel frameshift deletion mutation (c.422_428del, p.Leu142Valfs∗11) in exon 7 of *COL4A5*. In pedigree 2, the 16-year-old male proband had elevated serum creatinine (309 *μ*mol/L) without extrarenal manifestations, while his mother only manifested with hematuria. A missense mutation (c.476G>T, p.Gly159Val) was found in exon 9 of the *COL4A5* gene. Neither of these mutations was present in the Exome Variant Server of the NHLBI-ESP database, nor was it found in the ExAC or 1000 Genomes databases. Through the literature review, it was found that male Chinese patients with X-linked AS carried *COL4A5* deletion or missense mutations had a more severe phenotype than female patients, particularly in proteinuria and impaired renal function. Compared to male patients with missense mutations, patients in whom deletion mutations were found were more likely to progress to ESRD (15.4% vs. 36.0%, *P* = 0.041). This study identified two novel *COL4A5* mutations in Chinese families with X-linked AS, expanded the mutational spectrum of the *COL4A5* gene, and presented findings that are significant for the screening and genetic diagnosis of AS.

## 1. Introduction

Encoded by the collagen type IV alpha-3 (*COL4A3*), alpha-4 (*COL4A4*), and alpha-5 (*COL4A5*) genes, Alport syndrome (AS) is a rare inherited renal disease caused by abnormalities of the *α*3, *α*4, or *α*5 chains in type IV collagen [1]. These genetic defects lead to inadequate structure and function of basement membranes in glomeruli, the cochlea, ocular lenses, and other organs. The typical clinical features of AS are progressive nephritis, including microscopic hematuria, gross hematuria, proteinuria, impaired renal function or end-stage renal disease (ESRD), neurosensory deafness, and, occasionally, ocular lesions [1, 2].

AS has three genetic forms: X-linked AS, autosomal recessive AS, and autosomal dominant AS. X-linked AS is the most common type, accounts for 80% of cases, and is caused by defective *α*5 chains in collagen IV. The autosomal recessive inheritance pathway accounts for approximately 15% of cases and is characterized by *COL4A3* and/or *COL4A4* allele mutations. Autosomal dominant AS is rare (only 5% cases), with the pathological phenotype being caused by heterozygous mutations in *COL4A3* or *COL4A4* [3–5].

In X-linked AS, hemizygous males show more severe clinical symptoms than heterozygous females, with approximately 60% and 90% of males reaching ESRD before the age of 30 and 40, respectively [6]. Studies have found that, depending on the type of mutation they carry, males have a full range of clinical symptoms [6^,^^7^], while females with the *COL4A5* mutation show a variety of phenotypes even within the same family [8]. At present, a large number of mutations have been found in X-linked AS patients. The most common forms include missense mutations (about 38.0%), followed by deletion mutations (estimated 15.9%) and splicing mutations (about 14.9%) [6]. While the majority of pathogenic variants have been reported, the genotype-phenotype associations have not been studied. To date, the functional consequences of missense and deletion mutations in patients with X-linked AS have not been clarified.

This study reports novel *COL4A5* deletion (c.422_428del) and missense mutations (c.476G>T) found in Chinese families with X-linked AS. By reviewing the literature, this study is also aimed at investigating the typical clinical features of deletion and missense mutations in Chinese patients with X-linked AS.

## 2. Materials and Methods

### 2.1. Subjects

**Figure 1:**
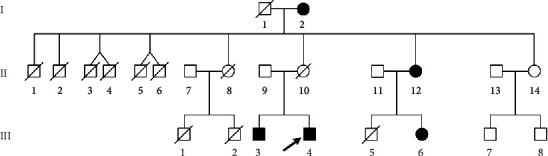
Pedigree 1 of the Alport syndrome family. Black-filled symbols represent patients carrying the c.422_428del (p.Leu142Valfs∗11) deletion mutation. The proband is marked with a black arrow.

The proband (III-4) in pedigree 1, a 31-year-old male, was admitted with ESRD to the department of nephrology at the First Affiliated Hospital of Jinan University in March 2019. Several years before admission, he presented with obvious hearing loss. Furthermore, the patient had a positive family history of kidney disease. Clinical information of the patient's family members was collected during interviews. This included age, gender, symptoms, previous history of disease, and positive test results. Blood samples from the proband and eight other family members were collected for genetic screening. The kidney function of participants was followed up 1 year later (Figure 1). The laboratory data were measured using a 7180 Biochemical Automatic Analyzer (Hitachi, Tokyo, Japan).

**Figure 2:**
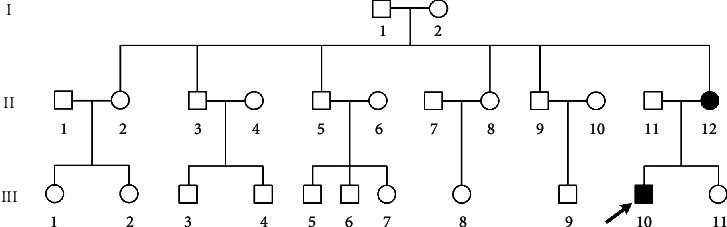
Pedigree 2 of the Alport syndrome family. Black-filled symbols represent patients carrying the c.476G>T (p.Gly159Val) missense mutation. The proband is marked with a black arrow.

The 16-year-old male proband (III-10) in pedigree 2 visited the nephrology outpatient department at the First Affiliated Hospital of Jinan University in September 2014. He had proteinuria at 1 year of age, while his mother (II-12) had microhematuria for a long period of time. The clinical information of the patient's family members was collected, and blood samples from the proband and his parents were collected for genetic screening (Figure 2).

This study was performed in accordance with the Declaration of Helsinki. Written informed consent was obtained from all participants before enrolment.

### 2.2. Whole Exome Sequencing (WES)

Genomic DNA was extracted from peripheral blood using a QIAamp DNA Blood Mini Kit (Qiagen China Co., Ltd., Shanghai, China) according to the manufacturer's instructions. The probands in pedigrees 1 and 2 were subjected to exome sequencing. All exon sequences were captured by Agilent SureSelect version 4 (Agilent Technologies, Santa Clara, CA, United States) according to the manufacturer's protocols. Captured DNA libraries were sequenced on an Illumina HiSeq X Ten platform according to the manufacturer's instructions for paired-end 150 bp reads.

### 2.3. Genetic Analysis

Paired-end reads were aligned to the NCBI build37 (hg19) database using BWA, while duplicated reads were marked by Picard [9]. SNVs and indels were detected by SAMtools and an in-house filter pipeline. Annovar [10] was used for annotation. Common polymorphisms were excluded based on their associated allele frequencies in the 1000 Genomes (ftp://ftp-trace.ncbi. http://nih.gov/1000genomes/ftp/), Exome Aggregation Consortium (ExAC, http://exac.broadinstitute.org/), and National Heart, Lung, and Blood Institute esp6500si (NHLBI, http://evs.gs.washington.edu/EVS/) databases. The assessment of the deleterious effects of the variants was conducted using multiple tools, including MutationTaster (http://www.mutationtaster.org/), SIFT, PolyPhen, and CADD in the annotation. Variants were also annotated with their clinical status of disease on the basis of the Human Gene Mutation database (HGMD, http://www.hgmd.org). Possible AS-associated variants were determined against the following factors: (i) rarity or absence in the three genome databases, (ii) the variation which was expected to have a drastic effect on the protein (nonsense mutation, frameshift mutation, mutations at splice sites, or missense mutations that were highly conserved among species), and (iii) the variation that was predicted to be pathogenic.

### 2.4. Mutation Validation

For candidate variant validation and pedigree analysis, polymerase chain reaction (PCR) and Sanger sequencing was performed. The PCR primers were designed using regions that were 500 bp up- and downstream from the site of interest and at least 25 bp sequence of each primer. Primers flanking the candidate loci were designed against the Human Genome reference genomic sequences from GenBank in NCBI and synthesized by Invitrogen (Shanghai, China). PCR amplification was carried out in an ABI 9700 Thermal Cycler (Applied Biosystems, Foster City, CA, United States) and the PCR products directly sequenced on an ABI PRISM 3730 automated sequencer (Applied Biosystems). Results were analyzed against *COL4A5* sequences (Entrez GeneID: 3508) retrieved from the University of California Santa Cruz Genome Browser (http://genome.ucsc.edu/).

### 2.5. Literature Comparison

To comprehensively review the pathogenicity of deletion and missense mutations in Chinese patients with X-linked AS, searches for primary studies were conducted on MEDLINE (PubMed) on May 15, 2020. Mesh terms “Alport syndrome or Alport's syndrome” and “X-linked or X linked or X linkage” were applied for the literature search. All full-text case reports and articles written in English and involving affected Chinese families were included. Thirteen papers describing the *COL4A5* deletion or missense mutations in Chinese families with X-linked AS were found [11–23]. Available clinical information and genetic data, including age at symptom onset, gender, urine test, kidney function, extrarenal symptoms, and type of gene mutation, were tabulated and analyzed.

### 2.6. Statistical Analyses

Continuous variables are presented as the mean ± standard deviation (SD), and categorical variables are expressed as frequency and percentage. Student's *t*-test or equivalent nonparametric test was used to compare continuous variables between groups, where appropriate. Differences between categorical variables were analyzed using a chi-square test or double-tailed Fisher's exact test. All values are two-tailed, and *P* < 0.05 was considered statistically significant. Data were analyzed using IBM SPSS Statistics version 25.0 for Windows (IBM, Armonk, NY, USA).

## 3. Results

### 3.1. Clinical Features of the AS Families

On admission, the proband (III-4) in pedigree 1 had severely impaired renal function, with serum urea of 49.9 mmol/L, serum creatinine (Scr) of 1793 *μ*mol/L, hemoglobin counts of 48.1 g/L, serum potassium of 4.95 mmol/L, and serum calcium concentrations of 1.16 mmol/L and consequently received emergency hemodialysis. In subsequent investigations, both the proband and his brother (III-3, 33-year-old) had microscopic hematuria, proteinuria (about 1–2 g/d), and typical extrarenal symptoms (binaural sensorineural hearing loss and ocular lesions). His brother had elevated Scr (516 *μ*mol/L), refused to undergo a renal biopsy, and began hemodialysis 1 year later. His father (II-9) was healthy and asymptomatic, while his mother (II-10) had died more than a decade prior of unknown causes. There were three male cousins (III-1, III-2, and III-3) who received hemodialysis, and all died from ESRD between 18 and 25 years of age. The affected female family members (I-2, II-12, and III-6) had microscopic hematuria, but no other symptoms. All six maternal uncles (II-1, II-2, II-3, II-4, II-5, and II-6) had died at a young age, but exact details relating to cause of death were not available (Table 1, Figure 1).

**Figure 3:**
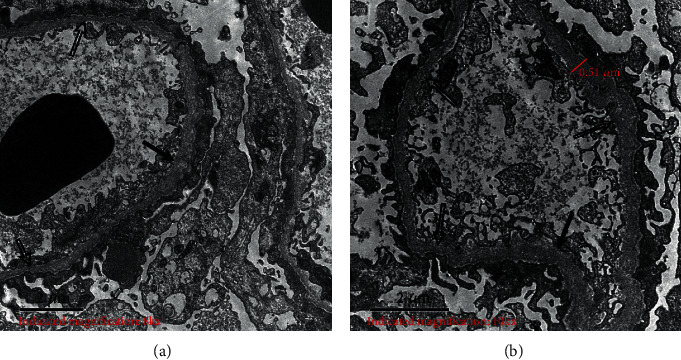
(a, b) Electron microscopy of the renal biopsy in the proband (III-10) shows that the thickness of the glomerular basement membrane varies (200–600 nm) and that the segmentary basement membrane was serrated. Some regions were torn and the cobweb structure altered. (The solid arrows represent thickening and hollow arrows represent thinning).

In pedigree 2, the proband (III-10) had proteinuria at the age of 1 year and presented with hematuria and proteinuria, without impaired renal function, hearing loss, or ocular lesions when he visited our outpatient department for the first time in 2014. Light microscopy by renal biopsy showed mesangial proliferative lesions in the glomerular segment with obvious interstitial foam cell infiltration. Electron microscopy revealed that the basement membrane was of variable thickness (200–600 nm). The segmentary basement membrane was serrated, and the dense layer was thickened, while some regions were torn and the cobweb structure altered. Substantial fusion of cellular processes occurred in podocytes (Figure 3). After 6 years of follow-up, he had elevated Scr levels (309 *μ*mol/L), but lacked extrarenal manifestations. His mother (II-12) presented with only hematuria, while his father (II-11), one aunt (II-8), and one uncle (II-9) were in good health (Table 2, Figure 2). The remaining family members refused to undergo genetic, urine, and blood testing.

### 3.2. Identification of Novel Mutations in *COL4A5* Gene

**Figure 4:**
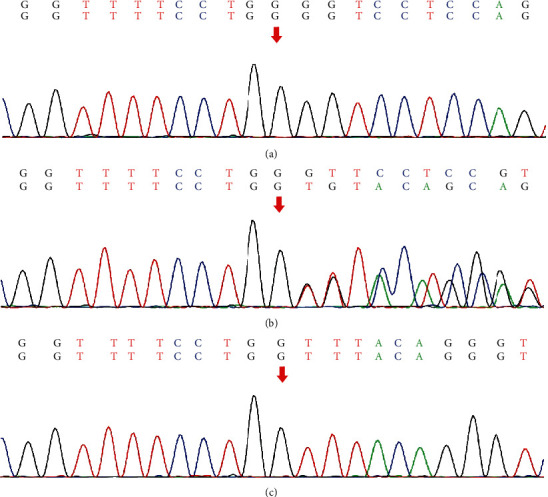
(a) Sequence of the hemizygous c.422_428del variant (III-3, III-4). (b) Sequence of the heterozygous c.422_428del variant (I-2, II-12, and III-6). (c) Sequence of unaffected individuals (II-9, II-14, III-7, and III-8).

Through WES and Sanger sequencing, a novel deletion mutation (c.422_428del) was identified in exon 7 of the *COL4A5* gene which was located on the X chromosome in pedigree 1. This deletion mutation resulted in the formation of a significantly truncated (p.Leu142Valfs∗11) COL4A5 protein, which was shortened from 1686 amino acids to 152 amino acids; 10 of which were aberrant residues. ClinVar database (https://www.ncbi.nlm.gov/clinvar/) analysis showed that this variant was followed by 52 terminating variants, all of which were pathogenic variants (PVS). This mutation was predicted by MutationTaster to cause nonsense-mediated mRNA decay (NMD), resulting in amino acid sequence and splice site changes. Protein features might, therefore, be affected. This hemizygous variant was found in both the proband (III-4) and his brother (III-3), while the heterozygous variant was detected in three affected female family members (I-2, II-12, and III-6). It was not found in the proband's father (II-9) and three unaffected individuals (II-14, III-7, and III-8; Figure 4). The c.422_428del variant cosegregated with the phenotype of the family. This mutation was not present in the Exome Variant Server of the NHLBI-ESP database, nor was it found in the ExAC, 1000 Genomes, and HGMD databases. According to the variant interpretation guidelines of the American College of Medical Genetics and Genomics (ACMG) [24], the c.422_428del (p.Leu142Valfs∗11) variant was classified as the pathogenic variant in this pedigree when PVS1, PM2, PP1, PP3, and PP4 criteria were included.

**Figure 5:**
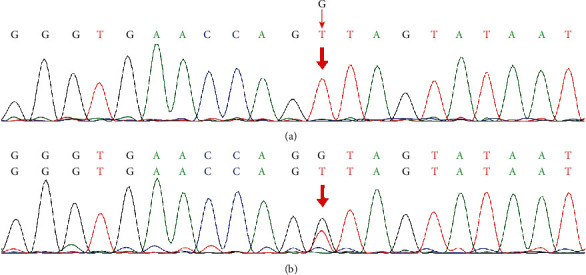
(a) Sequence of the hemizygous c.476G>T variant (III-10). (b) Sequence of the heterozygous c.476G>T variant (II-12).

A hemizygous missense mutation in exon 9 of *COL4A5*, c.476G>T, was found in the proband (III-10) represented in pedigree 2, resulting in the change of the 159th amino acid of the encoded protein from Gly to Val (p.Gly159Val). The heterozygous variant was detected in his mother (II-12), and the variant was not found in the proband's father (II-11, Figure 5). The variant and renal disorder was cosegregated in the family. According to the MutationTaster prediction, this mutation will cause changes to the amino acid sequence and splice site, thereby affecting protein features. This variant was not found in the Exome Variant Server of the NHLBI-ESP database and ExAC or the 1000 Genomes databases, but is a known disease-causing mutation in the HGMD database (HGMD CD113181). According to the ACMG standards and guidelines for variant interpretation [24], the c.476G>T variant in this pedigree was weighted as “PM2, PP1, PP2, PP3, and PP4” and classified as a variant of “likely pathogenic.”

### 3.3. Literature Review

A comprehensive review on deletion or missense mutations in the *COL4A5* gene in the Chinese population with X-linked AS was implemented. Data from this study and 13 published studies were included, thereby representing 141 affected participants among 88 Chinese families [11–23]. Twenty-four deletion mutations were discovered in 24 Chinese families involving 43 affected patients when including data from this study and nine published papers. The mean age of symptom onset was 9.1 ± 6.0 years. All patients had microscopic hematuria, 84.6% had proteinuria, while only 36.9% of males manifested gross hematuria. Sixty-three missense mutations were found in 64 Chinese families involving 98 affected patients from nine published papers. The mean age of symptom onset was 20.6 ± 13.8 years, which was significantly older than that observed with deletion mutations (*P* < 0.001). Nearly all patients had microscopic hematuria, 81.1% had proteinuria, while only 22.6% had gross hematuria (Table 3).

Male Chinese patients with X-linked AS had more severe phenotypes than female patients, especially regarding proteinuria and impaired renal function. More than half of the male patients had impaired renal function, which was significantly higher than that in female patients (18.9%, *P* < 0.001). Compared to male patients who carried missense mutations, those that carried deletion mutation were more likely progressed to ESRD (15.4% vs. 36.0%, *P* = 0.041). About half of the male participants with a deletion mutation had hearing loss and approximately a quarter showed ocular lesions. Comparatively, one-third of the male participants with a missense mutation experienced hearing loss and only 15.8% showed ocular lesions (Table 4).

## 4. Discussion

In the present study, a novel deletion mutation (c.422_428del) was identified in exon 7 of *COL4A5* using WES in a Chinese family with X-linked AS, a finding which was validated by Sanger sequencing. This frameshift deletion mutation produced a significantly truncated (p.Leu142Valfs∗11) COL4A5 protein product constituting 10 aberrant residues and only 152 amino acids. After this variant, ClinVar database had recorded 52 pathogenic terminating variants. This mutation was predicted to be “disease causing” by MutationTaster, causing NMD and changes in the amino acid sequence and splice sites and might affect protein features. The c.422_428del variant was cosegregated with phenotype in pedigree 1. In another family, a missense mutation (c.476G>T, p.Gly159Val) resulting in the change of the 159th amino acid of the encoded protein from Gly to Val was found in exon 9 of *COL4A5*. MutationTaster predicted this mutation to be “disease causing,” resulting in changes to the amino acid sequence and splice sites. Therefore, the protein features might be affected by this mutation. The variant and renal disorder was cosegregated in this family. Neither mutation was present in the Exome Variant Server of the NHLBI-ESP, ExAC, or 1000 Genomes databases. According to the ACMG guidelines [24], the novel c.422_428del (p.Leu142Valfs∗11) variant was the pathogenic variant for the disorder, while the c.476G>T (p.Gly159Val) variant was classified as being a variant of “likely pathogenic” for AS. The literature review revealed that, compared to female patients, male Chinese patients with X-linked AS have more severe phenotypes, particularly in proteinuria and impaired renal function. Of the male patients who progressed to ESRD, 36.0% carried deletion mutations. This rate was higher than those that carried missense mutations (15.4%, *P* = 0.041).

AS is a monogenic nephropathy that results in familial hematuria, progressive renal failure, sensorineural hearing loss, and ocular anomalies. It is caused by defects in type IV collagen, which is the major and necessary structural component of basement membranes in glomeruli, cochlea, and ocular lenses [25]. There are six *α* chains (*α*1–*α*6) encoded by the collagen type IV alpha-1 through alpha-6 genes (*COL4A1*, *COL4A2*, *COL4A3*, *COL4A4*, *COL4A5*, and *COL4A6*). Each of these genes have a common primary structure which includes a 25-residue “7S” domain at the amino terminus, a collagenous domain of approximately 1400 Gly-X-Y repeats, and a noncollagenous (NC1) domain approximately 230 residues in length at the carboxyl terminus [26]. AS is caused by mutations in *COL4A3*, *COL4A4*, or *COL4A5*, which results in structural and functional defects of *α*3, *α*4, or *α*5 chains [27]. AS has been reported to be inherited in three patterns, with an estimated mutation frequency of 1/5000–1/10,000 [28]. Among live births, the estimated prevalence of AS is about 1 : 50,000 [29]. According to the United States Renal Data System, approximately 3% of children and 0.2% of adults with ESRD in America have been diagnosed with AS, while AS accounts for 0.8% of Chinese patients with kidney diseases [30, 31].

As the major form, X-linked AS is caused by mutations in *COL4A5* [3–5]. The *COL4A5* is located at Xq22, contains 51 exons, and encodes the 1685 amino acid residues that constitute the *α*5 chain of type IV collagen [12^,^^32^]. The *α*5 chain is composed of a 26-residue signaling peptide, a 1430-residue collagenous domain (exons 2–47) containing the characteristic Gly-X-Y-repeat sequence and 22 short noncollagenous interruptions, and a 229-residue carboxyl-terminal NC1 domain (exons 47–51) [32]. Due to various mutations, patients with X-linked AS have phenotypes that range from benign familial hematuria to ESRD that is occasionally accompanied with neurosensory deafness and ocular lesions [1^,^^12^^,^^33^]. Hemizygous male patients usually present with more severe phenotypes than heterozygous females and have a higher probability of developing ESRD and/or hearing loss [6^,^^8^]. In our first pedigree, five affected male patients between the ages of 18 and 31 suffered from ESRD, while two presented with hearing loss and ocular lesions. The three affected female patients only presented with microhematuria. Similarly, the male proband in pedigree 2 had progressive glomerulonephritis and renal failure without extrarenal symptom, while his mother manifested only microhematuria. It was furthermore found through the literature review that more than half of the male patients had impaired renal function, a value that is significantly higher than that observed in female patients (18.9%, *P* < 0.001). Heterozygous females have widely variable disease outcomes, ranging from normal urinalysis and renal function to progression to ESRD and deafness. There was a significant genotype-phenotype correlation in male patients, which was not observed in affected females. Even within the same family, phenotypes differed between females. Thus, genotype might not be the main determinant of phenotypic heterogeneity in X-linked AS females. Phenotypic differences between males and females were considered to be related to allelic heterogeneity and skewing of X chromosome inactivation [7]. In recent years, some studies have suggested that the severity of AS in heterozygous females might be affected by skewed X-inactivation patterns. Typically, females would inherit 50% of their active X-chromosomes from each of their parents. X chromosome inactivation was previously considered to be a stable and irreversible phenomenon. However, recent studies had indicated that the X chromosome inactivation rate might be skewed due to chance, X chromosome abnormalities, X-inactivation modifier genes, or selection advantages of mutants [7, 8, 34–36].

By July 2020, more than 1000 mutations had been identified in *COL4A5* according to the HGMD. These include large rearrangements and small mutations such as missense, deletion, insertion, nonsense, and splicing mutations [6]. Genotype-phenotype correlations between *COL4A5* mutations and X-linked AS have been widely described in a series of case studies and literature reports. According to a 2012 meta-analysis of genotype-phenotype correlation in X-linked AS by Gross et al., typical X-linked AS was suggested to be classified into three types, namely, severe, moderate-severe, and moderate. Severe cases are characterized by juvenile-onset (∼20 years of age) ESRD with 80% and 40% of cases involving hearing loss and ocular lesions, respectively. This type is considered to be associated with frameshift, premature stop, large rearrangements, donor splice site, and NC1 domain mutations. Moderate-severe patients are characterized by ESRD at ∼26 years of age with a lower probability of extrarenal symptoms. These cases are usually caused by nonglycine missense mutations, glycine substitutions involving exons 21–47, in-frame, and acceptor splice site mutations. Moderately affected individuals are characterized by late-onset ESRD (∼30 years of age), with 70% of the cases manifesting hearing loss and less than 30% having ocular lesions. These symptoms are related to glycine substitutions involving exons 1–20 [37–39]. This classification was consistent with our findings. In the first family, while the five male patients who presented with severe phenotypes harbored a novel deletion mutation (c.422_428del) in exon 7, the moderate-severe male proband in pedigree 2 who had elevated Scr (309 *μ*mol/L) without extrarenal manifestations at the age of 22 harbored a missense mutation (c.476G>T) in exon 9.

Currently, although considerable allelic heterogeneity has been reported, a large number of AS families still need to be analyzed in order to determine the genotype-phenotype correlation and characteristics due to the various mutation types. Jais et al. comprehensively reviewed the phenotypes of 401 male patients with X-linked AS from 195 families from the European Community Alport Syndrome Concerted Action [6]. The results showed that all male patients had hematuria and that the rate of progression to ESRD and hearing loss was mutation-dependent. The risk of developing ESRD before the age of 30 years was as high as 90% in male patients with large deletions and nonsense or frameshift mutations, but 70% and 50% in those with splicing or missense mutations, respectively. While roughly 60% of the patients with missense mutations exhibited hearing loss before the age of 30, this condition was observed in approximately 90% of the patients with other mutations. Although there are some literature reports concerning AS in the Chinese population, most of them are case studies. Observational studies that include a large sample size are still lacking. Through the literature review conducted on the clinical manifestations of deletion or missense mutation of *COL4A5* in Chinese families with X-linked AS, similar results to those reported in European patients were observed. Observational and follow-up studies with a larger sample size are however necessary for future research.

The present study has several advantages. First, this study identified two novel *COL4A5* mutations in Chinese families with X-linked AS and expanded the mutational spectrum of the *COL4A5* gene. Second, through a comprehensive literature review, the typical characteristics of *COL4A5* deletion and missense mutations in Chinese X-linked AS patients were explored. Third, novel mutations identified by WES may help to identify mutations in different genes that result in similar clinical presentations. This would assist in making the diagnosis of AS more accurate. The limitations of this study include the lack of genetic data of the proband's mother and renal biopsy data of the proband in pedigree 1 and the lack of clinical data of other family members in pedigree 2. Additionally, the sample size from the literature review was small, and the statistical power of the gender subgroup analysis was low. The c.476G>T (p.Gly159Val) variant was classified as “likely pathogenic” in pedigree 2 according to the ACMG criteria; however, the proband's kidney biopsy indicated typical pathological manifestations of AS, and WES only found this mutation. Finally, as an observational study, the exact relationship between genotype and phenotype in X-linked AS could not be firmly determined. A study involving a large sample size and follow-ups are thus needed to explore genotype-phenotype correlation.

In conclusion, this study identified novel *COL4A5* deletion and missense mutations in Chinese families with X-linked AS. This expands the mutational spectrum of *COL4A5* and is significant for the screening and genetic diagnosis of AS. Observational and follow-up studies with larger sample sizes are however needed to explore genotype-phenotype correlation among Chinese populations with X-linked AS.

## Figures and Tables

**Table 1:** Clinical and laboratory features of participants harboring the *COL4A5* c.422_428del (p.Leu142Valfs∗11) variant in pedigree 1.

Subjects	Gender	Age (years)	Urine test			Renal function			Extrarenal symptoms		Follow-up^b^			Mutation status
			MH	GH	Proteinuria	BUN	Scr	Age at ESRD	Hearing loss	Ocular lesions	BUN	Scr	Age at ESRD	
I-2	F	90	Y	N	N	4.6	51	N	N	N	4.5	52	N	Heterozygous
II-9	M	60	N	N	N	7.5	79	N	N	N	7.6	81	N	Wild homozygous
II-12	F	60	Y	N	N	4.5	52	N	N	N	4.9	60	N	Heterozygous
II-14	F	56	N	N	N	5.4	57	N	N	N	5.3	55	N	Wild homozygous
III-1	M	18^a^	—	—		—	—	18	—	—	—	—	—	—
III-2	M	21^a^	—	—	—	—	—	21	—	—	—	—	—	—
III-3	M	33	Y	N	Y	19.8	516	N	Y	Y	36.8	1180	34	Hemizygous
III-4	M	31	Y	N	Y	49.9	1793	31	Y	Y	30.4	1075	31	Hemizygous
III-5	M	25^a^	—	—	—	—	—	25	N	—	—	—	—	—
III-6	F	26	Y	N	N	5.7	61	N	N	N	5.1	52	N	Heterozygous
III-7	M	22	N	N	N	5.9	73	N	N	N	5.6	68	N	Wild homozygous
III-8	M	20	N	N	N	5.8	76	N	N	N	6.1	79	N	Wild homozygous

BUN: blood urea nitrogen (mmol/L); ESRD: end-stage renal disease; F: female; GH: gross hematuria; HD: hemodialysis; M: male; MH: microscopic hematuria; N: no; Scr: serum creatinine (*μ*mol/L), reference value, 46–103 *μ*mol/L; Y: yes. ^a^The age of death. ^b^Follow-up results one year after gene detection. — indicates that the value is not applicable.

**Table 2:** Clinical and laboratory features of participants harboring the *COL4A5* c.476G>T (p.Gly159Val) variant in pedigree 2.

Subjects	Gender	Age (years)	Urine test			Renal function			Extrarenal symptoms		Follow-up^**b**^			Mutation status
			MH	GH	Proteinuria	BUN	Scr	Age at ESRD	Hearing loss	Ocular lesions	BUN	Scr	Age at ESRD	
II-8	F	46	N	N	N	5.9	70	N	N	N	NE	NE	N	NE
II-9	M	43	N	N	N	6.3	82	N	N	N	NE	NE	N	NE
II-11	M	42	N	N	N	6.1	75	N	N	N	6.1	76	N	Wild homozygous
II-12	F	41	Y	N	N	7.1	80	N	N	N	7.0	82	N	Heterozygous
III-10	M	16^a^	Y	N	Y	7.1	87	N	N	N	12.9	309	N	Hemizygous

BUN: blood urea nitrogen (mmol/L); ESRD: end-stage renal disease; F: female; GH: gross hematuria; HD: hemodialysis; M: male; MH: microscopic hematuria; N: no; NE: not examined; Scr: serum creatinine (*μ*mol/L), reference value, 46–103 *μ*mol/L; Y: yes. ^a^Age of onset of III-10 was one year old. ^b^Follow-up results of six years after gene detection.

**Table 3:** Summary of clinical features among affected Chinese families with *COL4A5* deletion or missense mutations.

	Deletion mutation	Missense mutation	Total
Number of reports^a^	9	9	13
Pedigree	24	64	88
Participants	43	98	141
Mutations/variants	24	63	87
Number of involving exon	26	33	37
Gender (male : female)	26 : 17	57 : 41	83 : 58
Age of onset	9.1 ± 6.0 (*n* = 19)	20.6 ± 13.8 (*n* = 64)^**b**^	18.0 ± 13.3 (*n* = 83)
Age at study	21.6 ± 18.3 (*n* = 42)	25.6 ± 16.1 (*n* = 97)	24.4 ± 16.8 (*n* = 139)
Microscopic hematuria	100% (39/39)	97.9% (94/96)	98.5% (133/135)
Gross hematuria	26.9% (7/26)	22.6% (12/53)	24.1% (19/79)
Proteinuria	84.6% (33/39)	81.1% (77/95)	82.1% (110/134)
Impaired renal function	40.5% (17/42)	41.8% (38/91)	41.4% (55/133)
ESRD	26.2% (11/42)	10.0% (9/90) ^**c**^	15.2% (20/132)
Age at ESRD	24.5 ± 7.2	30.8 ± 10.9	27.4 ± 9.4
Hearing loss	41.7% (15/36)	31.1% (23/74)	34.5% (38/110)
Ocular lesions	21.9% (7/32)	13.6% (8/59)	16.5% (15/91)

The categorical variable was expressed as % (*n*/*N*), where *n* indicates the number of positive observations and *N* indicates the total number of indicators observed. ESRD: end-stage renal disease. ^a^Not included in this study. ^b^Compared to the deletion mutation group, *P* < 0.001. ^c^Compared to the deletion mutation group, *P* = 0.016.

**Table 4:** Summary of clinical features among affected Chinese families, stratified according to gender, with *COL4A5* deletion or missense mutations.

	Male			Female		
	Deletion mutation	Missense mutation	Total	Deletion mutation	Missense mutation	Total
Microscopic hematuria	100% (23/23)	98.2% (54/55)	98.7% (77/78)	100% (16/16)	97.6% (40/41)	98.2% (56/57)
Gross hematuria	42.9% (6/14)	25.9% (7/27)	31.7% (13/41)	8.3% (1/12)	19.2% (5/26)	15.8% (6/38)
Proteinuria	100% (23/23)	92.9% (52/56)	94.9% (75/79)	62.5% (10/16)	64.1% (25/39)	63.6% (35/55)^b^
Impaired renal function	52.0% (13/25)	57.4% (31/54)	55.7% (44/79)	23.5% (4/17)	18.9% (7/37)	20.4% (11/54)^c^
ESRD	36.0% (9/25)	15.4% (8/52)^a^	22.1% (17/77)	11.8% (2/17)	2.6% (1/38)	5.5% (3/55)^d^
Age at ESRD	23.8 ± 6.7	27.8 ± 6.5	25.6 ± 6.7	—	—	—
Hearing loss	52.4% (11/21)	36.0% (18/50)	40.8% (29/71)	26.7% (4/15)	20.8% (5/24)	23.1% (9/39)
Ocular lesions	23.5% (4/17)	15.8% (6/38)	18.2% (10/55)	20.0% (3/15)	9.5% (2/21)	13.9% (3/36)

The categorical variable was expressed as % (*n*/*N*), where *n* indicates the number of positive observations and *N* indicates the total number of indicators observed. ESRD: end-stage renal disease. ^a^Compared to the deletion mutation group, *P* = 0.041. ^b,c,d^Compared to the male group, *P* < 0.001, *P* < 0.001, and *P* = 0.009, respectively. — indicates that the value is not applicable.

## Data Availability

The data used to support the findings of this study are available from the corresponding author upon request.
